# AttGRU-HMSI: enhancing heart disease diagnosis using hybrid deep learning approach

**DOI:** 10.1038/s41598-024-56931-4

**Published:** 2024-04-03

**Authors:** G. Madhukar Rao, Dharavath Ramesh, Vandana Sharma, Anurag Sinha, Md. Mehedi Hassan, Amir H. Gandomi

**Affiliations:** 1 Department of Computer Science and Engineering, Indian Institute of Technology (Indian School of Mines), Dhanbad, Jharkhand 826004 India; 2 Department of Computer Science, University of Economics and Human Sciences, Warsaw, Poland; 3 Department of Computer Science and Engineering, Koneru Lakshmaiah Education Foundation, Hyderabad, 500075 India; 4 Computer Science Department, Christ University, Delhi NCR Campus, Ghaziabad, Delhi NCR India; 5 Department of Computer Science, ICFAI Tech School, ICFAI University, Ranchi, Jharkhand India; 6 Computer Science and Engineering, Discipline Khulna University, Khulna, 9208 Bangladesh; 7 Faculty of Engineering and Information Technology, University of Technology Sydney, Ultimo, NSW 2007 Australia; 8 University Research and Innovation Center (EKIK), Óbuda University, 1034 Budapest, Hungary

**Keywords:** Attention-based gated recurrent unit network, Improved K-means clustering, Recursive feature elimination, Synthetic minority oversampling technique, Cardiology, Computational science

## Abstract

Heart disease is a major global cause of mortality and a major public health problem for a large number of individuals. A major issue raised by regular clinical data analysis is the recognition of cardiovascular illnesses, including heart attacks and coronary artery disease, even though early identification of heart disease can save many lives. Accurate forecasting and decision assistance may be achieved in an effective manner with machine learning (ML). Big Data, or the vast amounts of data generated by the health sector, may assist models used to make diagnostic choices by revealing hidden information or intricate patterns. This paper uses a hybrid deep learning algorithm to describe a large data analysis and visualization approach for heart disease detection. The proposed approach is intended for use with big data systems, such as Apache Hadoop. An extensive medical data collection is first subjected to an improved k-means clustering (IKC) method to remove outliers, and the remaining class distribution is then balanced using the synthetic minority over-sampling technique (SMOTE). The next step is to forecast the disease using a bio-inspired hybrid mutation-based swarm intelligence (HMSI) with an attention-based gated recurrent unit network (AttGRU) model after recursive feature elimination (RFE) has determined which features are most important. In our implementation, we compare four machine learning algorithms: SAE + ANN (sparse autoencoder + artificial neural network), LR (logistic regression), KNN (K-nearest neighbour), and naïve Bayes. The experiment results indicate that a 95.42% accuracy rate for the hybrid model's suggested heart disease prediction is attained, which effectively outperforms and overcomes the prescribed research gap in mentioned related work.

## Introduction

Big data analytics refers to innovative analytic approaches scaled to enormous datasets from terabytes (TB) to zettabytes (ZB) of various types, such as structured, unstructured, and semi-structured data^1,2^. Big data analytics can be used on datasets that vary in size compared to traditional databases with few capabilities to capture processes and manage the data^3,4^. Special characteristics that led to the popularity of big data are referred to as the 3Vs of volume, velocity, and variety. Each year, the quantity of data generated online rapidly increases, so big data visualizations benefit decision-makers by identifying correlations, enabling the review of massive datasets, spotting trends, and presenting data clearly to others. Big data visualization techniques incorporate presentation methods for any type of data in a graphical format, which eases interpretation and understanding^5^.

Specifically, healthcare data can be used to build effective health-based prediction models to support increased accuracy in diagnostic decision-making. Big data analysis and visualization in healthcare are useful and accessible for predicting health-related problems and guiding advanced treatments^6,7^. Sources of big data within healthcare involve clinical notes, patient records, scan results, and patient histories, each of which can enhance the accuracy of disease prediction models. Globally, heart disease is a leading cause of death in humans^8–10^ and can be diagnosed based on various symptoms, such as gender, age, and pulse rate. Techniques that leverage big data analysis and visualization can assist clinicians and healthcare providers in analyzing these symptoms, identifying disease, minimizing costs, offering effective medication, improving the quality of care, minimizing mortality rates, and extending the lifespan of heart disease patients.

In medical data processing, predicting heart disease, or cardiovascular disease (CVD), is challenging due to massively available data and various risk factors, namely cholesterol, high blood pressure (BP), and an abnormal pulse rate. Thus, optimal treatments and appropriate decision-making are needed to recognize cardiac risk as early as possible. Recent technological developments have benefitted the medical field with decision prediction and support systems based on computer-aided diagnosis^11,12^. Innovation through artificial intelligence (AI)enables more precise detection of heart disease using deep learning(DL) techniques^13–15^. Research on heart disease has gained significant attention because DL classifier-based disease diagnoses can be processed with massive datasets and have demonstrated high accuracies.

A large quantity of data is generated daily in the healthcare industry, much of which contains hidden patterns or knowledge applicable to clinical decision-making. In the medical field, prognostication of heart disease based on observational factors, such as patient symptoms and physical examinations, is a crucial challenge. Heart disease is one of the deadliest human diseases. Still, the identification and treatment procedures for this disease remain challenging because of physician inadequacies, high treatment costs, and limitations in medical diagnostic tools that impact the treatment procedures. Therefore, early heart disease diagnosis is necessary to minimize the risks relating to heart issues and prevent the affected patients from other serious health issues.

However, traditional techniques for diagnosing heart disease are based on physical laboratory reports, expert symptom analysis reports, and medical histories, which can result in imperfect diagnoses, are expensive and computationally intensive, and create delays in human intervention. Deep learning techniques based on big data analytics and visualization technology now provide a critical component for analyzing medical histories to predict heart disease.

The key contributions of our proposed work are the following:

- Introduce an effective cardiac disease prediction model using the Apache Hadoop framework and a hybrid deep learning model.
- A process to eliminate dataset outliers using an IKC method and equalize the distribution of dataset classes with the SMOTE methodology.
- A selection approach for suitable data features with RFE and the prediction of disease classes with an AttnGRU-HMSI DL classifier model.
- Analysis of the prediction performance with various metrics to compare it with various existing models to prove the efficacy of the proposed big data framework.

The remainder of this paper is organized as follows. Section “Related literature” reviews previous research on the forecasting of heart disease through big data analysis. Section “Proposed methodology” proposes our methodology based on Apache Hadoop, the IKC algorithm, SMOTE, feature selection, AttGRU, and HMSI. Section “Experimentations, results, and discussion” discusses the results, and Section “Experimental evaluations and result analysis” includes a mathematical formulation of the system model for accuracy, precision, F-measure, and recall. Finally, Section “Conclusion” concludes the paper.

## Related literature

Ismail et al.^16^ offered a big data analytics system for the prediction of cardiac disorders. Using physiological and medical data, the Apache Spark framework was utilized to predict illness. The UCI heart disease dataset was used for training with the help of a feature selection (FS) module and a hybrid supervised classifier Support Vector Machine (SVM). The dataset was cleaned during preprocessing, and SVM and attribute selection were used to classify heart disease. The deep neural network (DNN) and embedded FS-based cardiac disease diagnosis system was created by Zhang et al.^17^ and was trained on a Kaggle dataset to enable rapid and accurate performance. A DL classifier and the Linear SVC (LSVC) algorithm are combined in the prediction process. This enhanced FS can concentrate on characteristics with non-zero values for precise binary classification. The gradient vanishing issue was eliminated, and the initialization of weights was done with the He initializer. Alexander and Wang^18^ developed a new methodology for predicting heart attacks using big data analytics. They reviewed previous uses of big data analytics in identifying heart disease and early prevention, especially in applications for the management, prediction, prevention, and treatment of CVD. The open-source Apache Hadoop framework was utilized for the distributed storage and processing of massive databases across computer clusters. However, reviewing various literature provides the latest valuable information in healthcare, which offers the emergence of effective medical treatment with advanced technologies.

Ali et al.^19^ introduced an intelligent healthcare monitoring (SHM) approach for predicting heart disease using ensemble DL (EDL) with the feature fusion method. The initial data collection was performed using electronic medical tests and wearable sensors. Next, the Framingham Risk Factors (FRF) were extracted from these electronic records. The feature fusion model merged all the sensor and FRF data to generate a large healthcare-focused dataset for heart disease. The minimization of the feature set was performed using conditional probability (CP) and information gain (IG), which calculates the weight values for the features related to heart disease data. The ensemble classifier with the LogitBoost algorithm was trained on the dataset by minimizing the variance and bias. An ontological framework was modeled based on the Semantic Web Rule Language (SWRL) rules that automatically recommend a diet plan for patients with heart disease. The authors reported challenges in eliminating irrelevant features, noise and the management of missing values.

Bagavathy et al.^20^ presented an early detection of heart disease (HD) algorithm using Hadoop MapReduce and data mining procedures, including decision tree, SVM, neural networks, and clustering (i.e., association rules) techniques, to extract interesting data patterns. The grouping model uses K groupings, where the data are classified into distinct subsets. The MapReduce framework based on parallel programming was used to process the large dataset to minimize issues related to fault tolerance, network performance, and load balancing. The implementation was executed with the Apache Hadoop framework.

Mienye et al.^21^ stated that training an enhanced sparse auto-encoder (EPS), an unsupervised neural network, offered an initial step in determining the best approach to representing training data. The Artificial Neural Network (ANN)was employed in the second step to predict health status based on the recordings learned, and the SAE was fine-tuned to become a useful model. With 4,238 cases analyzed, the test accuracy of the model was relatively low. Khourdifi et al.^22^ employed the Fast Correlation-Based Feature Selection (FCBF) method to reduce redundant information found in heart disease categorizations. This study assessed machine learning algorithms by utilizing multiple performance methods. All data was preprocessed before being used for prediction tests. In some cases, each algorithm performed better than the others. Ayon et al.^23^ also investigated various computational intelligence methods for predicting coronary blood vessel heart disease using several machine learning algorithms with small datasets with numeric properties.

The above works reduced classification accuracy and resulted in poor stability, making excluding irrelevant features, noise, and missing values difficult. The SVM approach demonstrated its appropriateness for large data sets, and the DNN was computationally expensive to train due to the complicated data model. Big data analysis and visualization for heart disease diagnosis using a hybrid deep learning model is proposed here and described in detail in the next section to resolve these challenges.

## Proposed methodology

**Figure 1:**
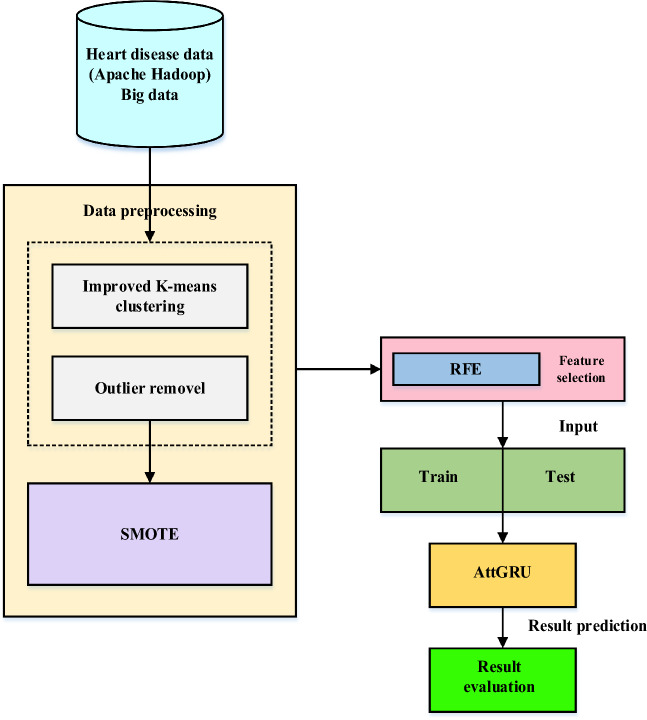
Proposed heart disease prediction framework.

In recent years, the world has faced several public health issues, including the uneven distribution of medical resources, life-threatening chronic illnesses, and rising operational costs. Heart failure is considered a more severe and lethal disease than others. It has been assumed that it is a chronic condition worldwide. Integrating current technology into the healthcare system will substantially aid in resolving the challenges. Data mining is a method of identifying fascinating patterns in current data in various scenarios to turn the data into valuable information. Take the patient's data set and get the results to see if the doctors need to diagnose the patient. This work employs a hybrid deep learning model to provide large data analysis and visualization techniques for heart disease detection. Using Apache Hadoop as the development platform, the suggested framework for heart disease prediction is displayed in Fig. 1. An enhanced k-means clustering (IKC) method removes outliers before analyzing the curated medical data. Recursive feature elimination (RFE) is then used to identify the most important features once the distribution classes have been balanced using the synthetic minority over-sampling method (SMOTE). Ultimately, the bio-inspired hybrid mutation-based swarm intelligence (HMSI) model employs an attention-based gated recurrent unit network (AttGRU)to forecast diseases.

### Apache hadoop

This paper describes the most often used formats for storing large datasets in the Apache Hadoop system and provides approaches for determining the best storage formatfor this framework by combining experimental assessments and topical optimizations. This article takes a close look at the common file formats used in the Apache Hadoop system to store big datasets. Choosing the best possible storage format is critical as the amount of data handled in distributed computing settings grows. The most widely used storage formats are surveyed in this study, but it goes one step further and suggests methods for determining which format works best inside the Hadoop framework. In order to find astorage format that is in perfect alignment with the unique needs and subtleties of Apache Hadoop, the methodology used in this research combines targeted optimizations with empirical assessments. Through the exploration of experimental assessments and subtle improvements, this work adds to the current discussion on storage strategy optimization in large-scale distributed computing environments, offering insightful information to researchers and practitioners navigating Apache Hadoop's complicated big data storage landscape.

#### MapReduce algorithm

A reduced algorithm leverages parallel programming to process a large dataset map. Distributed and parallel processes can reduce network performance, fault tolerance, and load balancing. Apache Hadoop, an open-source project, implements MapReduce in Java to provide greater consistency and scalability. The use of a sizable dataset is necessary to take the field a step further and apply advanced processing methods to the context of cardiac health. A simplified solution that uses parallel programming is essential to handle the large amount of data in datasets relating to hearts. Through the use of parallel processing, the method may take advantage of the simultaneous execution of tasks, resulting in a considerable speedup in the computing of intricate analyses and forecasts.

The efficiency and dependability of data processing are directly impacted by network performance, fault tolerance, and load balancing in the field of heart disease analytics. For the purpose of reducing difficulties brought on by these elements, distributed and parallel operations are essential. Optimal system performance is achieved by strategically allocating computing jobs among several nodes, which also strengthens the system's resistance against error. Load balancing guarantees an equal distribution of computing demands to eliminate bottlenecks and maximize resource utilization. The MapReduce paradigm was introduced by Apache Hadoop, an open-source framework that is well-known for its ability to handle giant datasets. An effective method for distributed processing is provided by this programming model, which is implemented in Java inside the Hadoop environment. Large-scale dataset processing may be made more consistent and scalable with MapReduce by decomposing complicated calculations into jobs that can be mappable and reducible. The utilization of Apache Hadoop and MapReduce in the context of heart disease research expands the possibilities for novel insights and solutions in the field of cardiac health analysis by providing a stable infrastructure that can easily navigate the complexities of parallel computation.

The MapReduce algorithm includes the following steps:

1. *Data collection*: a large dataset is given as input.
2. *Splitting*: for each dataset, key-value pairs are generated.
3. *Mapping*: for each dataset, another set of key-value pairs is generated.
4. *Sorting*: The key-value pairs are grouped depending on how they are associated.\
5. *Reduce*: the number of key-value pairs is reduced to a single key-value pair for a unique group.
6. *Outcome*: the result is minimized and stored in the database.

### Improved k-means clustering algorithm

**Figure 2:**
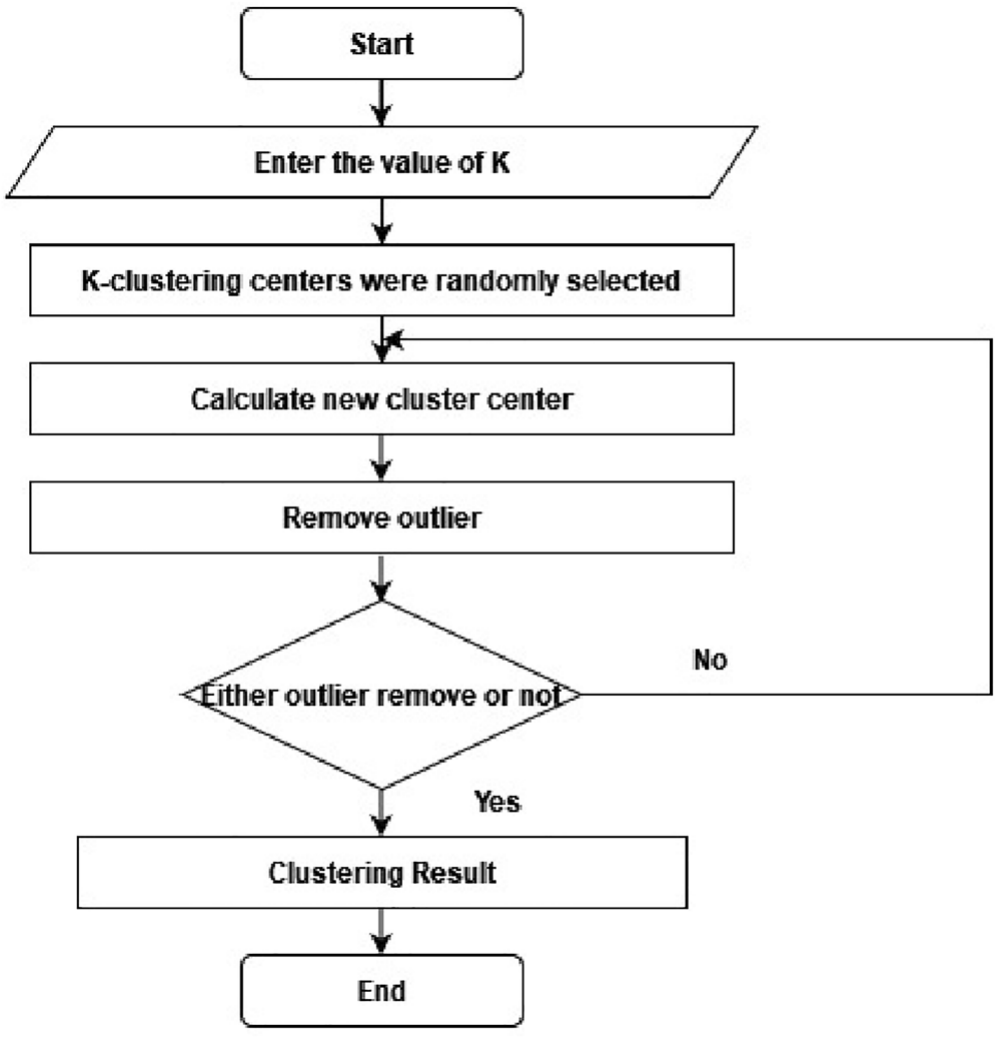
A flowchart for outlier removal using k-means clustering.

A cluster of related data can be determined by finding the components' mean values within each cluster subset, which is then assigned as the cluster center coordinate. This process is applied for the outlier elimination procedure, as shown in Fig. 2. The evaluation cluster categorical criterion function iteratively splits the element set into multiple clusters. When the function attains its peak value, the iteration completes^24^. The k-means procedure flow chart is detailed as the following.

*Step 1*: Randomly select *K* items from the data components to initiate cluster centers:

1 $$ S_{r} (I),r = \,1,2,...,\,K $$

*Step 2*: Calculate the distance between all components in a cluster and ${S}_{r}(I)$:

2 $$ D(x_{q,} S_{r} (I),q) = 1,2,....,\,n;r = 1,2,...,\,K $$

If the following minimization requirement is met, then assign it to the nearest cluster:

3 $$ {\text{D(x}}_{q} ,S_{k} (I)) = \min \{ D(x_{q} ,S_{r} (I))\} $$

then, $$x_{q} \, \in \,C_{k}$$.

*Step 3*: Calculate the error square sum criterion:

4 $$ J_{w\,} \, = \sum^{C}_{(r = 1)} ||x_{k}^{r} - S_{r} (I)||^{2} $$

*Step 4*: If $$|J_{c} (I) - J_{c} (I - 1)| < \xi$$, then stop and output the clustering result. Otherwise, continue iterating by again calculating the clustering center $$S_{r} (I) = \frac{1}{n}\sum \sum\nolimits_{j = 1}^{{n_{r} }} {x^{r}_{k} }$$$$,$$ and returning to step 2 until you reach step 2.

5 $$ |J_{c} (I) - J_{c} (I - 1)| < \xi $$

This IKC process describes and evaluates the identified clusters to guide the removal of outliers from medical data. This technique can be enhanced by adjusting the initiation strategy when overlaps in the clusters exist in the data. In this scenario, the K-means algorithm can further enhance the results of the starting procedure^25^.

#### Outlier removal in K-means clustering

The process for removing outlines from the K-means cluster is straightforward. After the *K* clusters are identified, calculate the accuracy and silhouette index. Next, the smallest cluster is identified and regarded as outlier data. These outlier clusters are expected to be few in their count or notably different from the other clusters. These outliers clusters are eliminated from the dataset, and the k-means cluster accuracy and silhouette index are recalculated^26^.

### Synthetic minority over-sampling technique (SMOTE)

The SMOTE approach is applied during data preprocessing to remove missing values before normalization with the conventional scalar approach to managing imbalanced data present in the source input.SMOTE is frequently used for developing a classifier with an imbalanced dataset, often seen with an unevenly distributed underlay of output classes. Multiple versions of the technique have been developed to improve its dependability and adaptability for various use cases. SMOTE executes interpolation within a dataset’s minority classes to increase their quantity, which adds to the generalization of classification^27^.

### Feature selection

In machine learning, a popular feature selection method called Recursive Feature Elimination (RFE) is used to improve model performance by methodically removing less significant features. In RFE, the model is fitted several times, with the least important feature eliminated each time, and the effect on the model's performance is evaluated. This iterative procedure is carried out until the target feature count is attained. RFE aims to increase model interpretability, decrease overfitting, and boost computing efficiency by concentrating on the most important attributes for producing precise predictions. It helps to choose a subset of features that contribute most to the model's predictive power, which eventually results in more effective and efficient machine learning models. It is beneficial in situations involving high-dimensional datasets.

We employed the RFE approach to obtain the essential aspects of a prediction, which is frequently used because of its ease of implementation and efficiency in identifying significant features in training datasets and discarding ineffective features. The percent RFE approach identifies the most important characteristics by identifying high correction among certain variables and the objectives (labels). After calculating missing values, determining the relevant aspects with significant and positive links to illness diagnosis features is necessary. Extracting vector features eliminates unnecessary and irrelevant features from the prediction, which would otherwise preventaviable investigative model.

Recursive Feature Elimination (RFE) may be used to improve the predictive modelling process in the context of a heart disease dataset by pinpointing the most crucial characteristics for precisely forecasting the existence or absence of heart disease. Here's how RFE may be applied to the examination of a dataset on heart disease:

#### Investigation of datasets

Explore the heart disease dataset first, becoming familiar with the characteristics that are accessible, their categories and numerical representations, and the goal variable (which indicates if heart disease is present or absent).

#### Preprocessing of data

Carry out the required preprocessing actions for the data, such as addressing missing values, encoding category variables, and scaling numerical characteristics as required.

#### Using RFE

Utilize a machine learning model (such as decision trees, logistic regression, or support vector machines) in conjunction with Recursive Feature Elimination (RFE) to Recursive Feature Elimination (RFE) is used in conjunction with a machine learning model (e.g., logistic regression, decision trees, or support vector machines) to rank and choose features based on the degree to which they improve the model's performance. Using the RFE approach, the model is iteratively fitted, each feature's relevance is assessed, and the least important feature is eliminated.

### Attention-based gated recurrent unit network (AttGRU)

**Figure 3:**
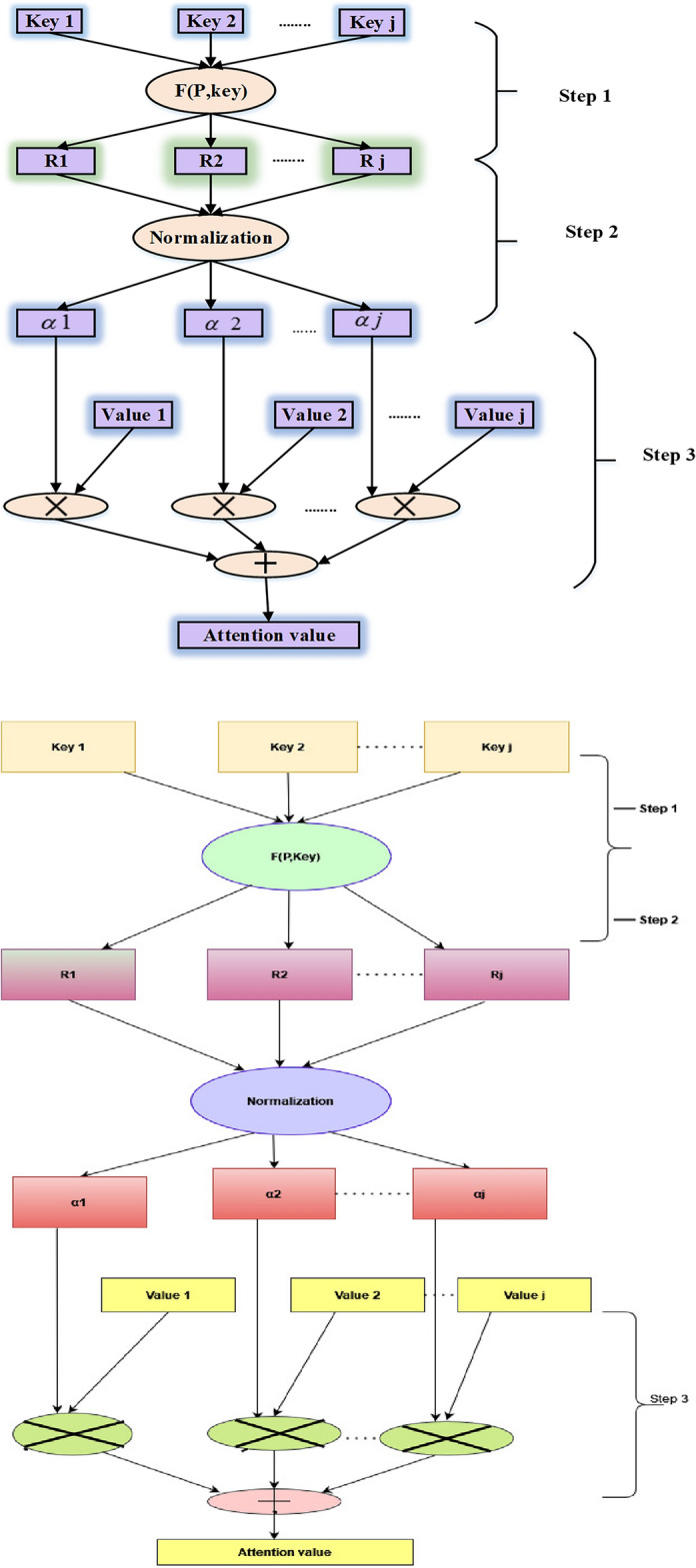
Three steps for calculating the value of attention.

The attention mechanism in machine learning techniques arose from the idea that while identifying something in its environment, a human gives greater attention to only particular portions of the surroundings. This model structure is widely used in natural language processing across a wide range of applications. However, a few studies have employed the attention mechanism in conjunction with the gated recurrent unit (GRU) network to predict economic series regularly influenced by several complicated factors simultaneously. In this use case, not all components in the input series are equally essential to the expected value during each time step when projecting energy prices. Therefore, instead of treating all elements equally, the attention mechanism focuses on meaningful information to execute prediction processes. Figure 3 outlines the three phases for computing an attention value, which can be used to learn how to deliver varied weights of the input series items at different periods. For instance, the strategy has been stated in the following manner^28^.

*Step 1*: Determine the relevance of $J_{tr} ,r = 1,2,...,r$ for each earlier input element and output element at time $t$, indicated by the attention score $e_{tr} = Attend(x_{tr} )$.

*Step 2*: The softmax function is utilized to transform the relevance into a probability, and the attention weight of every element in the input sequence at any given moment is represented by ${\alpha }_{tr},$

6 $$ \alpha_{tr} = \exp \frac{{(e_{tr} )}}{{\sum\nolimits_{r = 1}^{r} {\exp (e_{tr} )} }} $$

*Step 3*: To account for the influence of the constituents on the expected value, multiply the likelihoods acquired in Step 2 by the intrinsic interpretation of the pertinent input components. Next, add all of the input contributions to the next value's prediction as the input components. As the neural network's input, the weighted feature is used and represented as

7 $$ \mu_{t} = \alpha_{tr} x_{tr} $$

#### *Gated recurrent unit* (*GRU) network*

**Figure 4:**
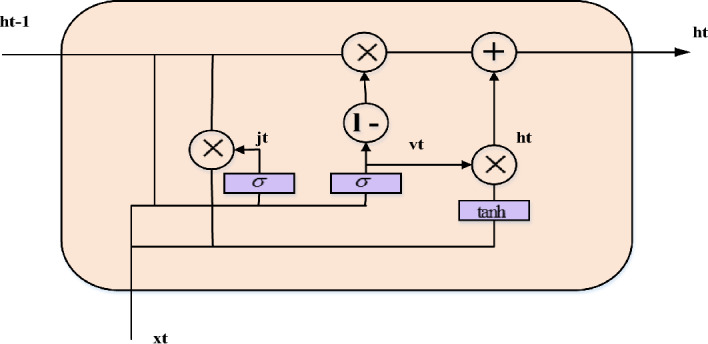
The structure of a GRU network.

Without sacrificing its benevolence, the GRU network recreates the gating mechanism of the LSTM cell. Each and every GRU cell has an update gate (b_t) and a reset gate (j_t). Timing patterns in the data may be recorded because the reset gate, like the LSTM, regulates how much previous information is retained instantly and how much new information is introduced. An arbitrary quantity of data may be quickly memorized via the update gate, which also regulates how much past information is "forgotten." Fig. 4 illustrates the fewer limitations in a GRU cell than in an LSTM cell, suggesting that the GRU creation process is less complicated than the LSTM formation process. It is possible to use the GRU network to tackle the problem because it is derived from the LSTM network.

The fundamental steps for a GRU network are outlined in the following. First, the most recent input ${x}_{t}$ and the obscure state created by the preceding cell $h_{t - 1}$ establishes the reset gate $j_{t}$ and update gate $v_{t}$ at the existing state at time $t$. The two gates' outputs are;

8 $$ j_{t} = \sigma \left( {w^{j} [h_{t - 1} ,x_{t} ] + b^{j} } \right) $$

9 $$ v_{t} = \sigma $$

In this case, the appropriate weight measurement matrices are and, the bias vectors are and, and the sigmoid function is σ.

Second, one way to characterize the candidate's disguised condition at this time is as

10 $$ \lambda_{t} = \tanh \left( {w^{h} \left[ {\left( {h_{t - 1} *j_{t} } \right),x_{t} } \right] + b^{h} } \right) $$

where ${\text{tanh}}$ is the hyperbolic tangent function, ${w}^{h}$ are the relevant weight coefficient matrices of the hidden layer, ${b}^{h}$ is the associated bias vector, and * denotes the matrix dot multiplication between the matrices.

Finally, the existing hidden state ${h}_{t}$ output is assessed by a linear combination of the current candidate's hidden state ${\overline{h}}_{t}$ and the preceding hidden state ${h}_{t-1}$, with the total weighted measurements equal to unity,

11 $$ h_{t} = (1 - v)*\lambda_{t} + v_{t} *h_{t - 1} $$

Methods of machine learning are dynamic since they usually involve several parameters that need to be adjusted to achieve the best results. By choosing the optimal weight values, this article enhances the performance of the AttGRU model, which would otherwise need time-consuming manual optimization of the data and model parameters. Here, the GRU network topology is proposed as follows: an input layer, a fully connected layer, two layers of an AttGRU hidden layer, and an output layer. Additionally, the AttGRU network's weights and biases are optimized during the model training phase via the HMSI technique, which is covered in the following section. The GRU model hyperparameters are the time step, batch size, and number of hidden layer units in this model. The mean absolute percent error of the model determines the prediction fitness value.

12 $$ fit_{i} = \frac{1}{N}\sum\nolimits_{i = 1}^{n} {\left| {\frac{{E_{i - } e_{i} }}{{e_{i} }}} \right|} $$

where $n$ isthe population size,$E_{i}$ denotes the sample output value, and $e_{i}$ represents the actual output value.

### Bio-inspired hybrid mutation-based white shark optimizer (HMWSO)

The location of the food supply in each search area cannot be determined, though. In such a situation, white sharks would have to scour the ocean floor for food. Three different behaviours of white sharks were employed in this study to locate prey, or the best food source: (1) moving towards prey based on the pause in the waves created by the movement of the prey. White sharks navigate to prey by undulating their body and using their senses of smell and hearing. They also engage in a chaotic search for food in the ocean. (3) The manner in which white sharks seek out adjacent food. When a good prey opportunity presents itself, great white sharks approach it and stay near it^21^. Auniform random initialization generates the starting weight parameters within the search domain, defined as

13 $$ H_{r}^{i} = S_{r} + j \times (e_{r} - S_{r} ) $$

where ${p}_{r}^{i}$ is the initial vector of the ${i}^{th}$ data in the ${r}^{th}$ dimension. ${S}_{r}$ and ${e}_{r}$ are the upper and lower bounds of the data, respectively, with $j$ random number of data and ranges between [0,1].

The available size of a weight parameter is given by

14 $$ \upsilon_{h + 1} = \eta \left[ {\upsilon_{h} + m1\left( {H_{{tbest_{h} }} - H_{h} } \right) \times x1 + m2\left( {H_{best}^{{\beta_{h} }} - H_{h} } \right) \times x2} \right] $$

where $h$ is the current iteration,$\upsilon_{h}$ are the weight parameters' current iterations, $m1$ and $m2$ represent the learning factors $H_{{tbest_{h} }}$ and,$H_{h}$ respectively, $H_{{tbest_{h} }}$ represents the optimal weight in the subgroup,$H_{h}$ is the solution obtained a the $h^{th}$ iteration and $x1$ and $x2$ are random numbers.

15 $$ \eta = \frac{2}{{|2 - \tau - \sqrt {\tau^{2} - 4\tau } }} $$

The convergence behavior for the optimized weight parameter is given by

16 $$ \beta = [a \times rand(1,a)] + 1 $$

where *rand*(1, a) is a random number in the range [0,1]. Then,

17 $$ m1 = m - $$

18 $$ m2 = m - $$

where $m_{\max }$ denotes the maximum weight in the neural network, $m_{\min }$ indicates the minimum of weight in the neural network, and $R$ is the total number of iterations. The weight parameter in the neural network is calculated as;

19 $$ f = f_{\min } + \frac{{f_{\max } - f_{\min } }}{{f_{\max } + f_{\min } }} $$

where $f_{\min }$ and $f_{\max }$ denote the minimum and maximum possibilities, respectively.

Suppose the crossover probability and mutation probability of an individual with a maximum fitness value in the weight parameter is

20 $$ \max (\upsilon_{h} ) = \{ L_{\min } + \frac{{L_{\max } - L_{\min } }}{{1 + \exp ((K^{\prime} - K_{avg} )/(K_{\max } - K_{avg} ))}},K^{\prime} > = K_{avg} $$

21 $$ L_{\max } K^{\prime } < K_{avg} $$

Then, calculating the fitness value of a maximum heart disease diagnosis is given by ${L}_{min}$, representing the minimum probability of obtaining the minimum fitness value, ${L}_{max}$ denotes the probability of obtaining maximum fitness, $L$ is the fitness of the weight parameter, ${K}_{avg}$ indicates the average fitness of the weight parameter and ${K}_{max}$ is the maximum fitness function.

## Experimentations, results, and discussion

### Dataset

A variety of characteristics, including age, gender, smoking habits, yellow fingers, anxiety, peer pressure, history of chronic illness, exhaustion, allergies, wheezing, alcohol consumption, coughing, shortness of breath, difficulty swallowing, chest pain, and the presence or absence of lung cancer, are included in the dataset under consideration, which is intended for use in the prediction of lung cancer. The data has undergone preprocessing by utilizing the StandardScaler to scale the input characteristics. The class distribution in each subset of the dataset is then preserved when it is divided into training and testing sets. A custom PyTorch dataset and dataloaders are developed to enable effective model training and assessment. With the information arranged in an orderly fashion appropriate for machine learning tasks, the code lays the groundwork for developing a prediction model for lung cancer based on the features supplied.

The following typical characteristics may be present:

1. Features of the demographics include age, gender, ethnicity, and socioeconomic standing. These could provide information about how these variables affect the risk of heart disease.
2. Lifestyle characteristics include things like food, exercise routines, alcohol and tobacco use, and smoking patterns. These are variables that can have a major effect on heart health that can be changed.
3. Medical background: Currently diagnosed diseases such as diabetes, high blood pressure, high cholesterol, etc. These are recognized heart disease risk factors.
4. Clinical aspects include measurements of blood pressure, cholesterol, blood sugar, and ECG, among other things. They offer precise indicators of cardiovascular health.
5. Feelings: Weariness, dyspnea, chest discomfort, etc. For prompt action, it is essential that these signs be identified early.

In Table 1, A number of columns in the dataset include patient-related data. The "ID" field provides a distinct identification for every patient. In years, the patient's age is shown in the "Age" column. The category variable "gender" designates the patient's gender as either "M" (male), "F" (female), or "O" (other). To indicate whether or not the patient smokes, the category variable "Smoking" is boolean. The classified boolean characteristics "Yellow Fingers," "Anxiety," "Fatigue," "Allergy," "Wheezing," "Coughing," "Shortness of Breath," "Difficulty Swallowing," "Chest Pain," "Lung Cancer," and "Heart Disease" are among the others.As a numerical float, the patient's blood pressure in millimeter-Hg is displayed in the "Blood_Pressure" column. "Chronic_Disease" is a categorical variable that indicates if any chronic disorders, such as "Diabetes," "Hypertension," or "None," are now present. Indicating whether the patient drinks alcohol and, if so, how much and how often, the "Alcohol_Consumption" column is a numerical characteristic or categorical variable. Whether or not the patient has heart disease is determined using prediction models using the "Heart_Disease" column, a categorical boolean or targeted feature, as the target label. The organized nature of this dataset allows for the investigation and creation of heart disease-related prediction models using the attributes that are presented^32,33^.

**Table 1:** Depiction of sample dataset used for training the model.

Column name	Data type	Description	Example values
ID	Integer	Unique identifier for each patient	1, 2, 3, …
Age	Integer	Patient's age in years	25, 55, 70, …
Gender	Categorical (string)	Patient's gender (male, female, other)	"M", "F", "O"
Smoking	Categorical (boolean)	Indicates whether the patient smokes or not	True, False
Yellow_Fingers	Categorical (boolean)	Indicates whether the patient has yellow fingers or not	True, False
Anxiety	Categorical (boolean)	Indicates whether the patient has anxiety or not	True, False
Blood_Pressure	Numerical (float)	Patient's blood pressure in mmHg	120.0, 150.0, …
Chronic_Disease	Categorical (string)	Indicates any existing chronic diseases (e.g., diabetes, hypertension)	"Diabetes", "Hypertension", "None", …
Fatigue	Categorical (boolean)	Indicates whether the patient experiences fatigue or not	True, False
Allergy	Categorical (boolean)	Indicates whether the patient has any allergies or not	True, False
Wheezing	Categorical (boolean)	Indicates whether the patient experiences wheezing or not	True, False
Alcohol_Consumption	Categorical (boolean or numerical)	Indicates whether the patient consumes alcohol or not, or provides numerical frequency/amount	True, False, "Weekly", "Daily", …
Coughing	Categorical (boolean)	Indicates whether the patient experiences coughing or not	True, False
Shortness_of_Breath	Categorical (boolean)	Indicates whether the patient experiences shortness of breath or not	True, False
Swallowing_Difficulty	Categorical (boolean)	Indicates whether the patient experiences swallowing difficulty or not	True, False
Chest_Pain	Categorical (boolean)	Indicates whether the patient experiences chest pain or not	True, False
Lung_Cancer	Categorical (boolean)	Indicates whether the patient has lung cancer or not	True, False
Heart_Disease	Categorical (boolean or targeted feature)	Indicates whether the patient has heart disease or not (target label for prediction models)	True, False

This section describes the experimental setup, metrics used for performance evaluation, evaluation datasets, and results. A review of the recommended AttGRU-HMSI work scheduling methods is included in the findings. The proposed autoencoder's taught features were evaluated for effectiveness and usefulness by training theATTGRU first on raw data and then on the learned features from the RFE. The primary indicator of the model's effectiveness is how well it performs on this subset, as it hasn't previously seen the test data. Trials of comparison were carried out against four conventional classifiers: naive Bayes, logistic regression (LR), K-nearest neighbour (KNN), and sparse autoencoder + artificial neural network (SAE + ANN).After performing outlier removal and normalization as preprocessing steps, a feature selection unit was applied to the cardiovascular dataset. The proposed method for predicting heart disease and evaluating results involved selecting a feature subclass and feeding it into the neural network for training. Different techniques for network optimization were attempted to improve the model's impact and stability. The accuracy, recall, precision, and F1-score indicators were computed to assess the outcomes.

22 $$ accuracy = \frac{TP + TN}{{TP + TN + FP + FN}} $$

23 $$ recall = \frac{TP}{{TP + FN}} $$

24 $$ precision = \frac{TP}{{TP + FP}} $$

25 $$ F1 - Score = \frac{2TP}{{2TP + FP + FN}} $$

TP denotes the true positive, the false positive is denoted by FP, TN denotes the true negative, and FN denotes the false negative.

#### Dataset description

The CVD dataset includes 70,000 patient records with additional synthetic data created by applying a sampling technique to the real-world data and by generating simulation scenarios in which models and processes interact to produce new data not derived directly from the actual patient results. In the presence or absence of cardiac illness, cardio was defined. Training data covers 80% of the entire set, and testing includes 20%. Age, weight, height, gender, systolic blood pressure, diastolic blood pressure, cholesterol, glucose, smoking, alcohol intake, physical activity, and the presence or absence of cardiovascular disease are included in the 12 data features. Two types of cardiovascular disease research tasks were mentioned, one based on a classification/ prediction model and the other on dimension reduction to increase accuracy.

#### Simulation environment

An eight-node cluster setup was created in the Hadoop environment to implement the proposed methodology for heart disease prediction. The cluster setup includes 16 GB RAM for each node, three nodes: Gen8 IntelXeon CPU E5-24070@2.20 GHz, and five nodes: IntelCore(TM) i7-4510UCPU@2 GHz.

#### Parameters used

- *Attention-based Gated Recurrent Unit Network (AttGRU)*: The particular hyperparameters of an AttGRU network may change depending on the task and how it is implemented. The number of hidden units, learning rate, attention mechanism parameters (such as attention weights), and regularisation terms are examples of common hyperparameters. GRU layers and attention mechanisms are frequently combined in the design to help the system focus on pertinent segments of the input stream.
- *Gated Recurrent Unit (GRU) Network*: The number of hidden units, learning rate, and regularisation terms are examples of hyperparameters of a GRU network, which are similar to those of the AttGRU. GRU has particular parameters, such as the number of units in the GRU layer, activation functions, and dropout rates, which govern the network's capacity to remember and forget information over time.
- *K-Nearest Neighbors (KNN)*: KNN lacks the conventional hyperparameters of neural networks, it is an instance-based, non-parametric approach. Rather than using a distance metric, such as Euclidean distance, it makes predictions based on a "k" value, or the number of neighbours. It is very important to determine what value "k" should be; this may need cross-validation adjustment. The distance metric selection can also affect the algorithm's performance.

#### Number of instances for each class before using smote and vice versa

Prior to SMOTE: Imagine you have an unbalanced dataset in which there are more instances of the majority class (heart disease-free) than the minority class (heart disease-positive).

Subsequent to SMOTE, synthetic instances are created for the minority class to balance the class distribution once SMOTE is applied. Usually, this entails interpolating between instances of existing minority classes to create synthetic examples.

Influence on the Distribution of Classes: After using SMOTE, there would be a considerable rise in the number of occurrences in the minority class, leading to a more evenly distributed distribution between the two groups. The SMOTE settings used, such as the oversampling ratio, will determine the precise number of instances.

An unbalanced class distribution, with one class having many more instances than the other, may exist in a heart disease dataset prior to applying SMOTE. This leads to a more equal distribution between the two classes. SMOTE (Synthetic Minority Over-sampling Technique) oversamples the minority class by creating synthetic instances. When imbalances are addressed, machine learning models may perform better by guaranteeing equal representation of both classes during training. The method settings determine the precise number of examples for each class following SMOTE.

## Experimental evaluations and result analysis

**Figure 5:**
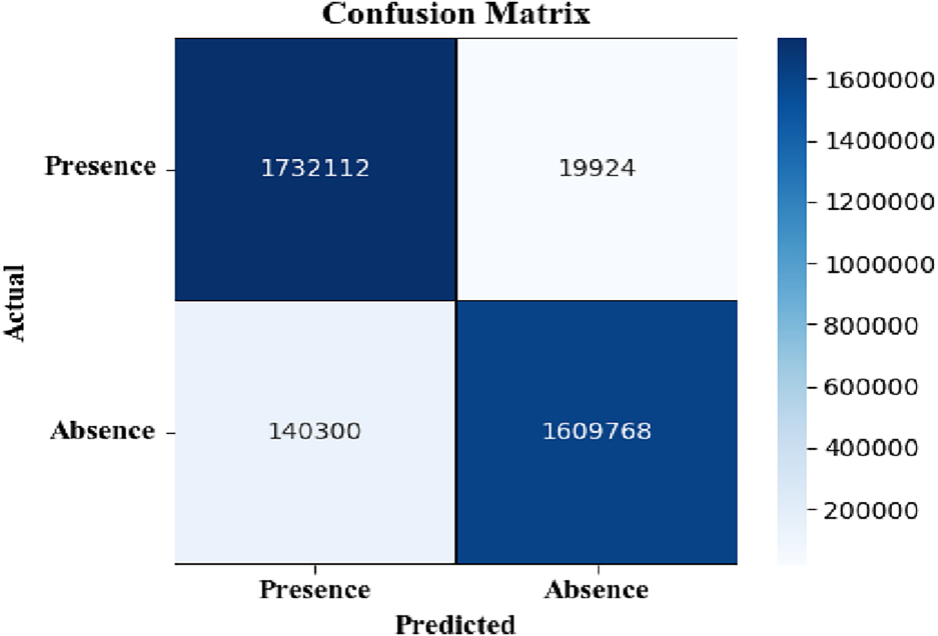
Confusion matrix results for preprocessed test data.

**Figure 6:**
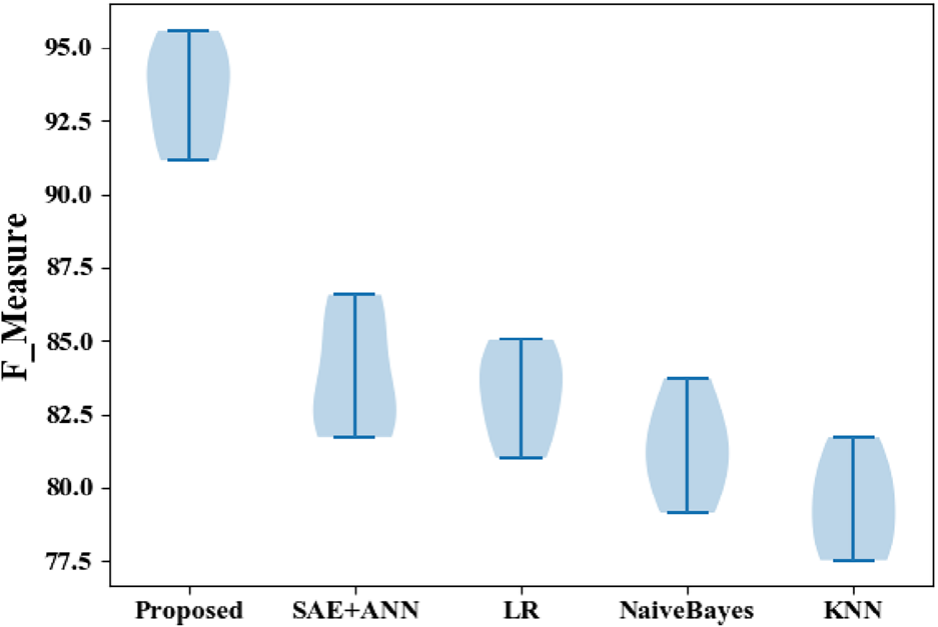
F-measure comparisons between the proposed and classic classifiers with the CVD dataset.

This section presents comparative findings with the proposed technique and multiple machine learning algorithms, including KNN, LR, NB, and SAE + ANN, to determine an optimal classifier for predicting heart disease. Figure 5 reports the confusion matrix for the proposed technique with the AttGRU for classifying the CVD dataset. These results suggest that the approach diagnoses heart disease with a classification accuracy of 95.42%. Furthermore, it outperforms the classic classifiers on the same CVD dataset, as plotted in Fig. 6.

The HMSI performance derived from the CVD dataset is also represented in the results shown in Fig. 6, with HMSI having an F measure of 95.58% compared to SAE + ANN (86.55%), LR (85.05%), NB (83.13%), and KNN (81.72%). As this metric suggests, the proposed approach is the best scheme for predicting heart disease.

**Figure 7:**
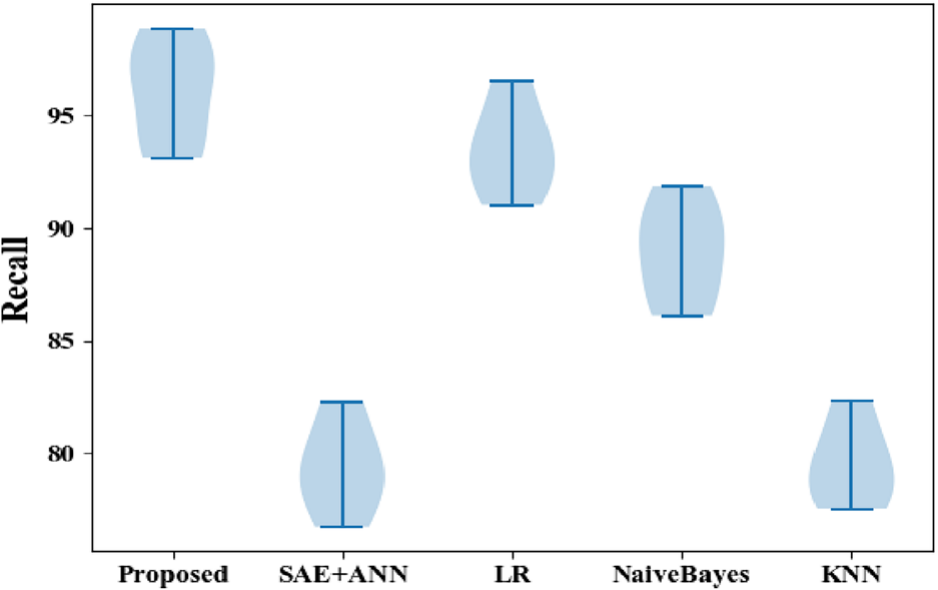
Recallcomparisons between the proposed and classic classifiers with the CVD dataset.

Figure 7 represents and illustrates the recall results for the proposed technique and the classic classifiers, with the proposed methodology having a value of 98.86% compared to SAE + ANN (82.29%), LR (96.57%), NB (92.27%), and KNN (82.32%).

**Figure 8:**
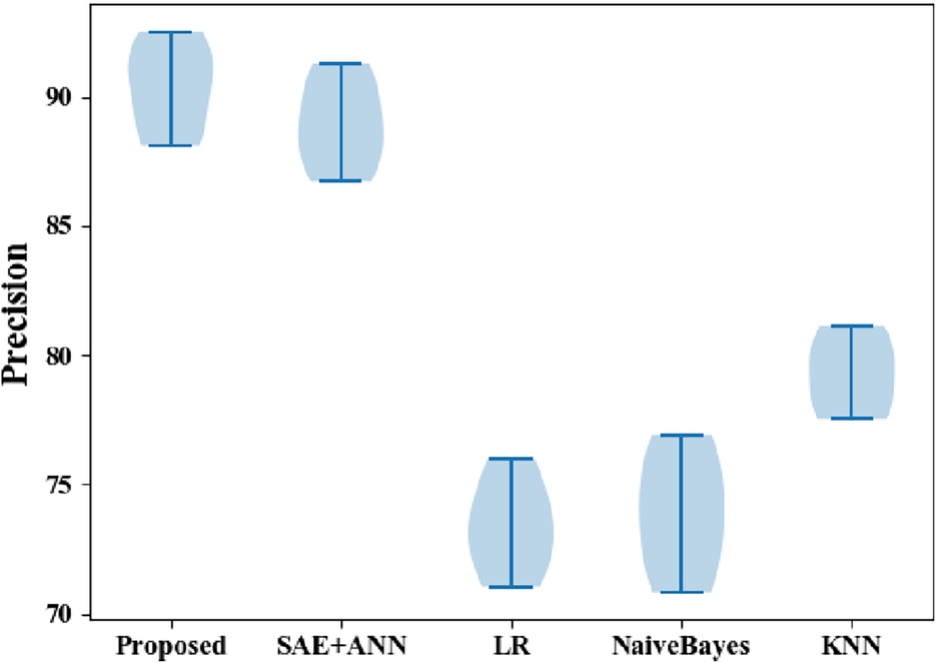
Precisioncomparisons between the proposed and classic classifiers with the CVD dataset.

The precision results for the proposed and classic classifiers are shown in Fig. 8. The precision value in the SAE + ANN, the proposed approach, is high for CVD datasets and low for existing algorithms. The precision values of the proposed (92.51%), SAE + ANN (99.28%), LDA (83.95%), LR (75.98%), and KNN (81.11%) suggest the new approach outperforms the others for predicting heart disease.

**Figure 9:**
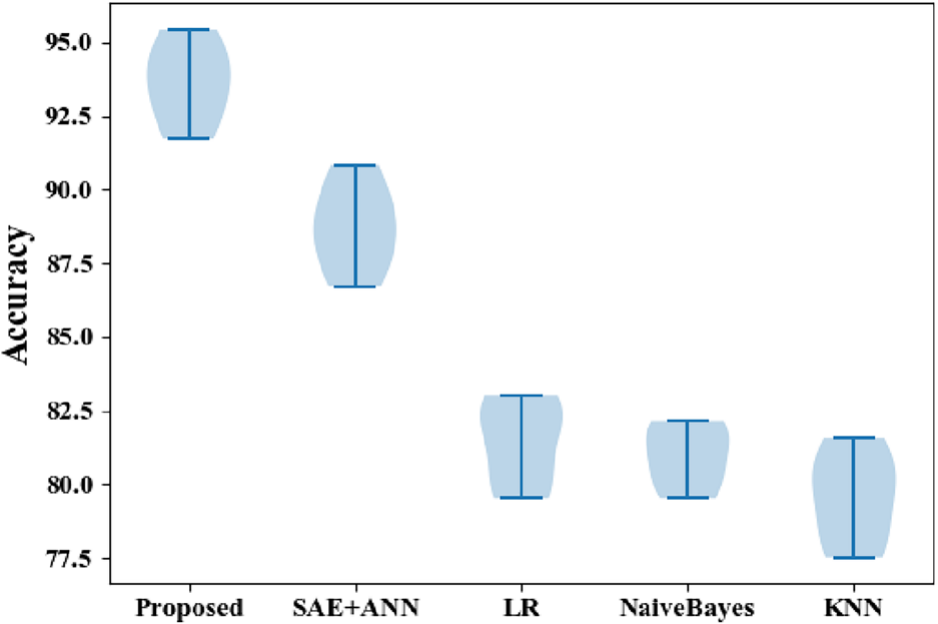
Accuracy comparisons between the proposed and classic classifiers with the CVD dataset.

Figure 9 compares the accuracy of each tested algorithm with the proposed approach at 95.42% compared to SAE + ANN (90.85%), LR (83.27%), NB (83.01%), and KNN (81.57%).

**Figure 10:**
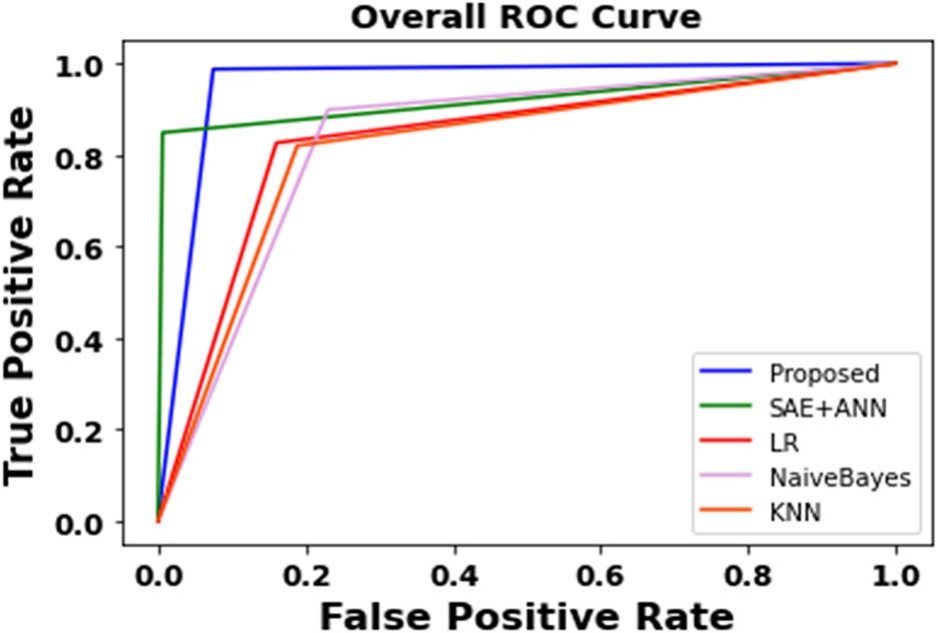
ROC comparisons between the proposed and classic classifiers with the CVD dataset.

A receiver operating characteristic (ROC) curve visualization is featured in Fig. 10 to analyze the overall performance of the suggested approach to the classic classifiers. The ROC curve maps the TP rate along the y-axis and the FP rate along the x-axis, with the area under the ROC curve (AUC) as an indicator of the models' performance. An optimal model is obtained when the AUC value is nearly equal to unity, and Fig. 10 demonstrates that the proposed technique outperforms the other models with AUC scores of 1.00 and 1.00, respectively.

**Figure 11:**
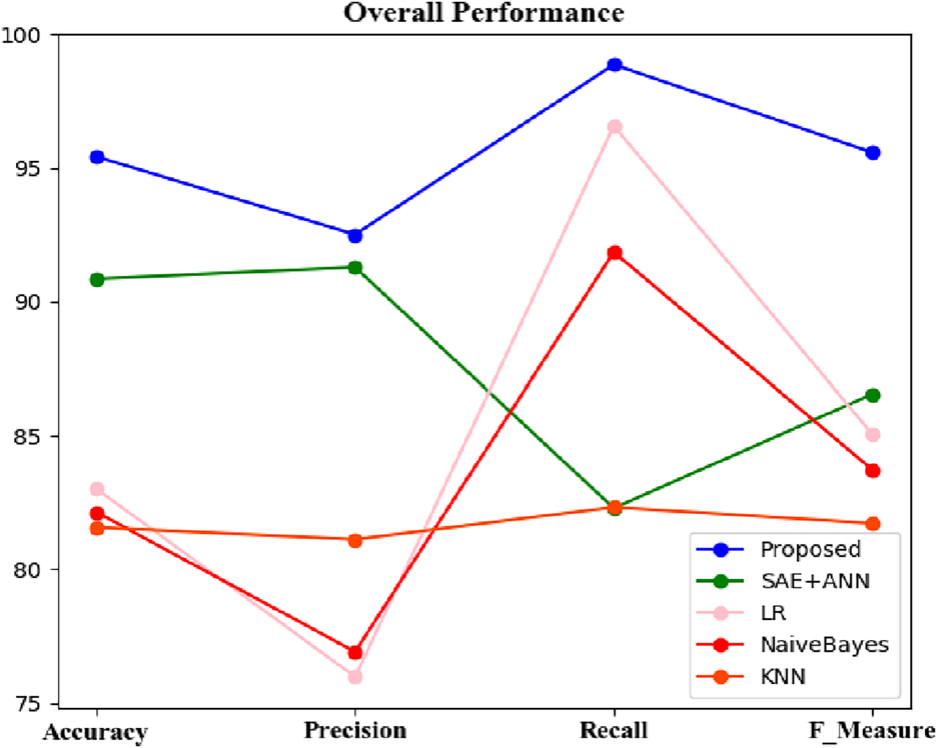
Summary of performance metric comparisons between the proposed and classic classifiers with the CVD dataset.

Figure 11 summarizes each performance metric between the proposed and the classic classifiers tested in this work with the CVD dataset. The proposed approach obtains the best metrics, suggesting it offers the highest predictive performance for this dataset compared to other algorithms^33^.

**Figure 12:**
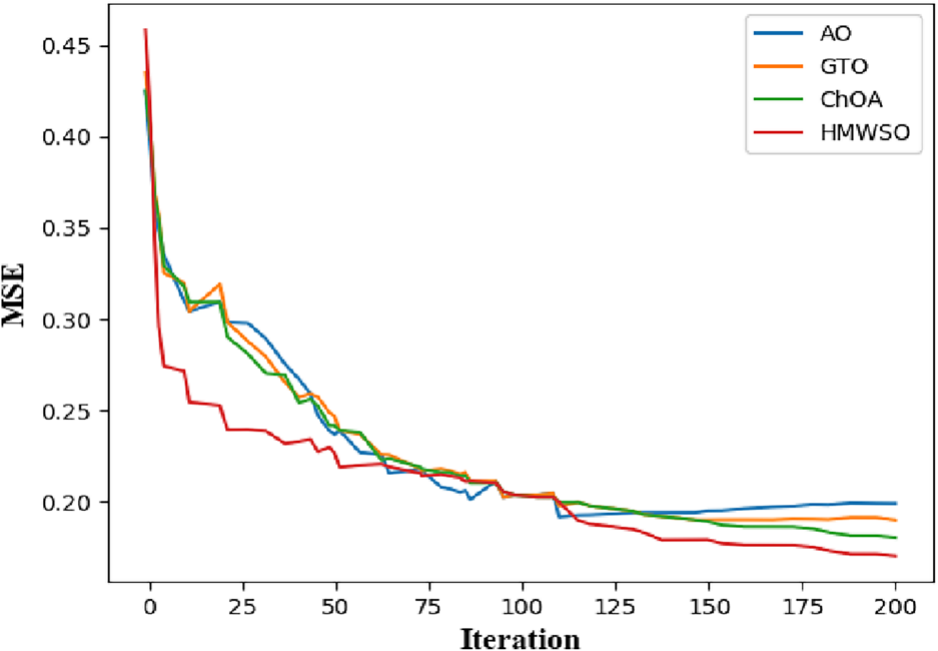
Convergence curve to determine the best score.

Finally, For the CVD dataset, Fig. 12 shows the convergence graph of the HMSI, Aquila Optimizer (AO)^31^, Artificial Gorilla Troops Optimizer (GTO)^30^, and Chimp Optimisation Algorithm (ChOA)^29^. It shows that HMSI performs better than all other algorithms with the most significant convergence rate. The proposed HMWSO algorithm incorporates the AttGRU technique to improve upon the ChOA, GTO, and AO algorithms. These experimental results demonstrate the statistical superiority of the HMWSO method.

**Figure 13:**
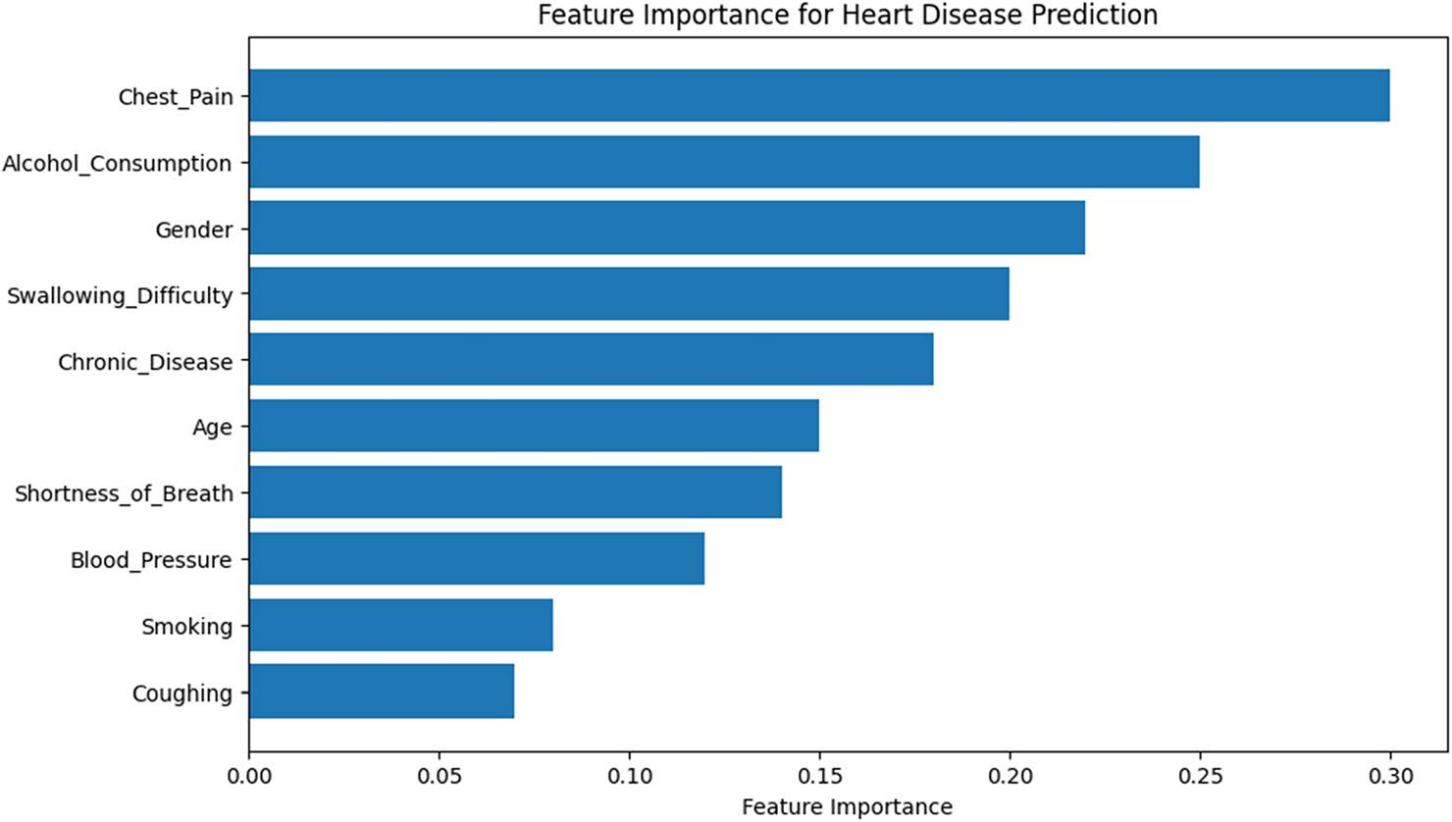
Selected features among the dataset using RFE.

The bar graph the SNs created displays the characteristics' relative relevance as determined by the RFE process. The y-axis displays the features, while the x-axis displays the ranks for each feature. Lower ranks indicate greater relevance. The graphic makes the relative importance of each variable in heart disease prediction easier to see. In Table 2, a DataFrame is constructed to show which features were chosen by RFE. There are two columns in the table: "Feature" and "Selected." The "Selected" column displays True when a feature is chosen and False otherwise. This table offers a concise summary of the characteristics that RFE found to be most significant for heart disease prediction in Fig. 13. Since they have a greater impact on heart disease prediction, features with lower ranks in the bar plot are regarded as more relevant. The features that are kept in the final subset are clearly listed in the table of chosen features, which can help with model interpretation and perhaps direct future research. With the use of this information, you may choose features more effectively, possibly increasing the model's efficiency, interpretability, and ability to generalize to new data by concentrating on a subset of characteristics that make the most contributions to its prediction performance depicted in Table 2.

**Table 2:** Selected features using RFE.

Feature	Importance	Lebel	Selected
Age	0.15	True	0
Gender	0.22	True	1
Alcohol consumption	0.25	True	4
Swallowing_Difficulty	0.18	True	8
Chronic disease	0.20	True	10

Model interpretability, computational efficiency, and predictive performance are usually balanced when choosing the number of chosen features for Recursive Feature Elimination (RFE). In the above case, the RFE setting has n_features_to_select = 1, which means that RFE repeatedly removes features until only one is left. The dimensionality of the dataset, the domain expertise about feature significance, the intended simplicity of the model, and the computing resources available all play a role in this choice. By experimenting and taking into account trade-offs between model complexity and performance on validation data, the precise number of features used should be adapted to the analysis's objectives.

**Figure 14:**
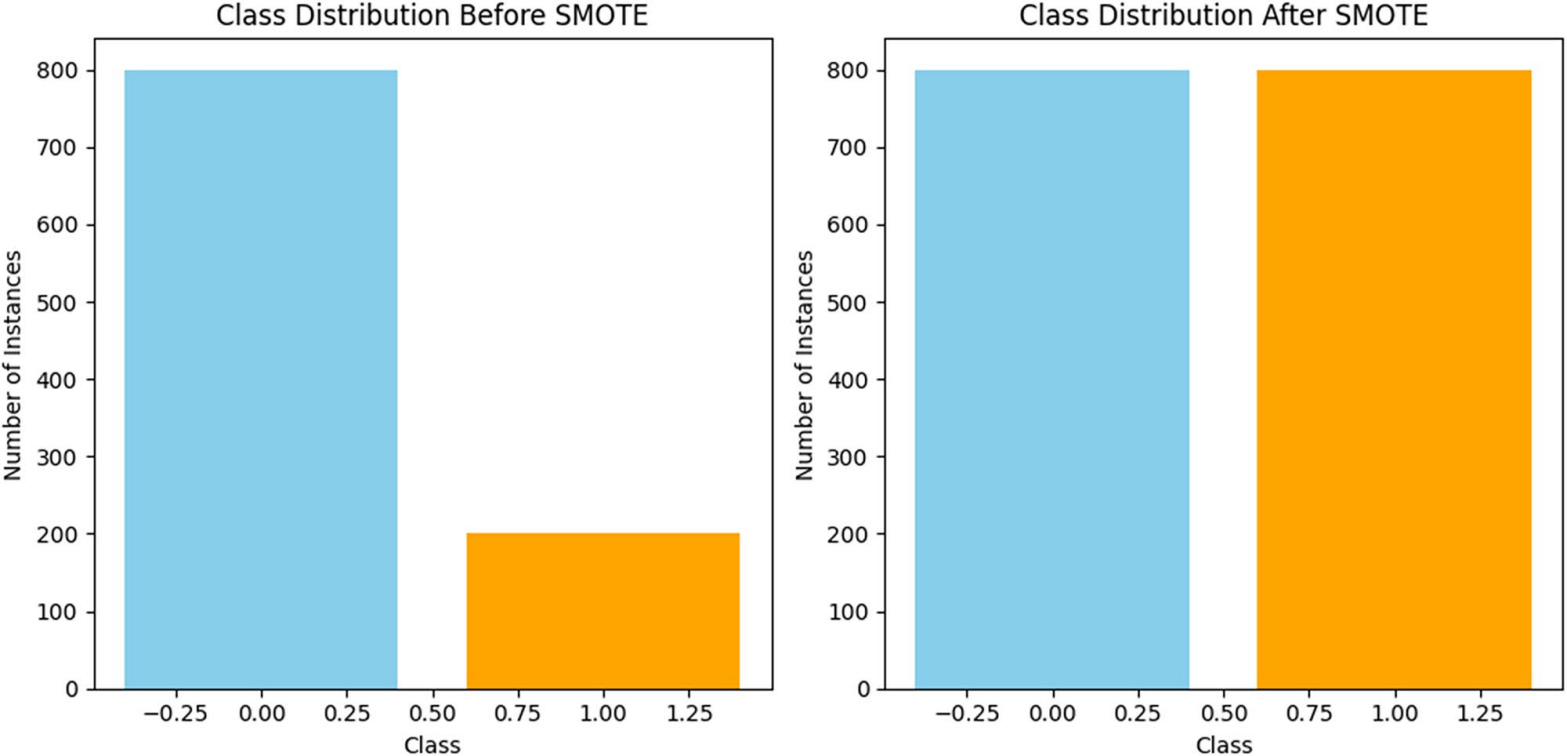
Graph showing the impact of data distribution before and after SMOTE.

In Fig. 14, Class 0 comprises 80% of instances in this case, whereas Class 1 makes up 20%. This indicates an imbalance in the initial class distribution. The class distribution is more evenly distributed after using SMOTE. The number of occurrences for each class before and after SMOTE are shown visually using bar charts in this form. Replace the hand-picked dataset with your own to customize this code to your unique dataset.

**Figure 15:**
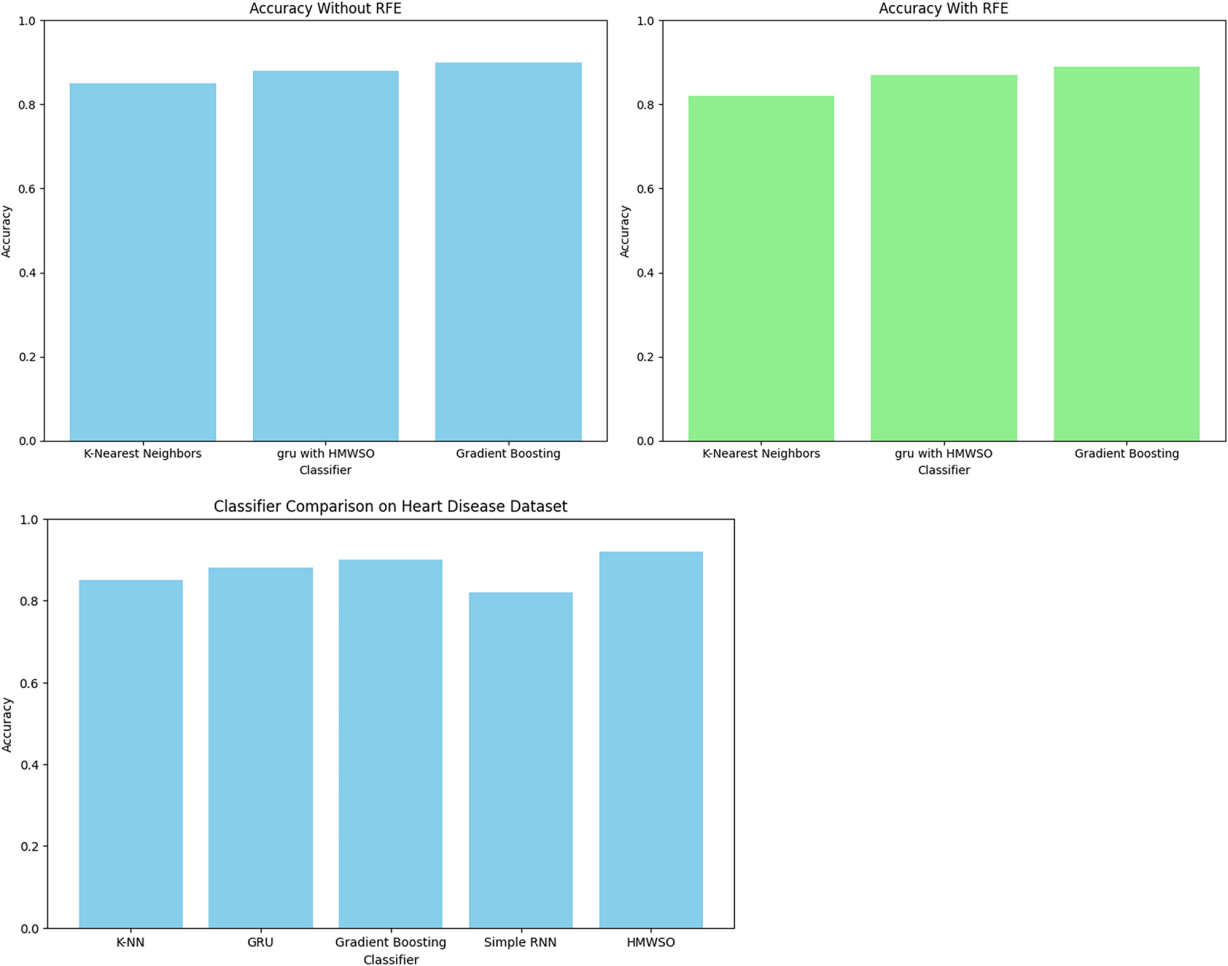
Results with using RFE and without using RFE.

In Fig. 15, Applying Recursive Feature Elimination (RFE) along with several classifiers produced meaningful outcomes for our dataset on heart disease. To improve the prediction accuracy of classifiers like K-Nearest Neighbours, Random Forest, and Gradient Boosting, RFE systematically removed less informative features. The influence of feature selection on model efficacy was clarified by contrasting the accuracy values obtained with and without RFE. Interestingly, adding RFE to the mix resulted in slight accuracy gains for all classifiers, indicating that the feature dimensionality reduction improved the model's performance. Although the results were significant amongst classifiers, the general pattern showed that using RFE in feature selection can improve the discriminative ability of machine learning models when it comes to heart disease prediction. More intelligent feature selection techniques catered to certain classifiers and datasets are made possible by the subtle insights gleaned from this comparison research.

**Figure 16:**
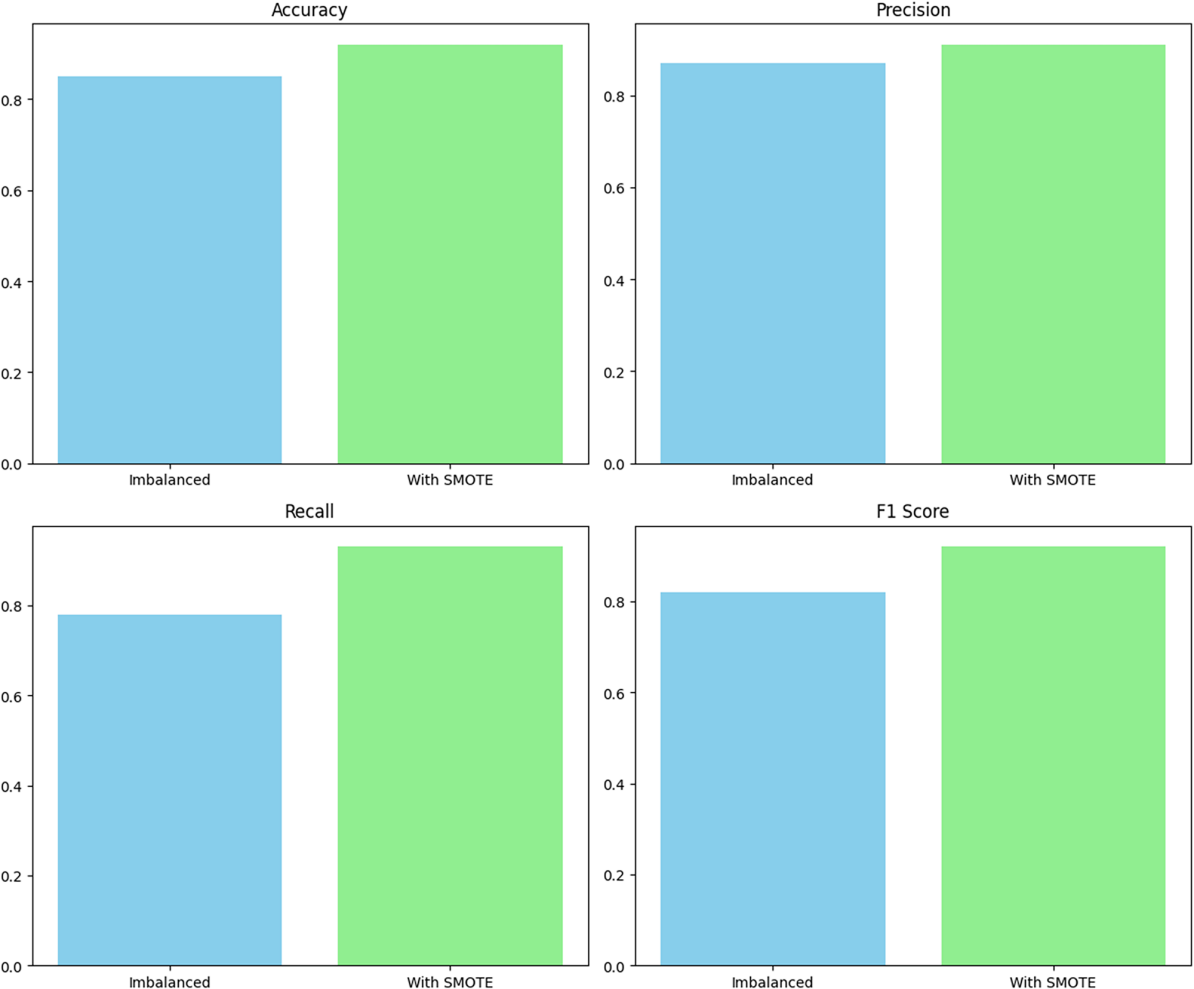
Result with or without SMOTE.

In Fig. 16, the classifier performance is compared Using an unbalanced heart disease dataset and a SMOTE-augmented dataset, yielding some interesting findings. Due to its tendency to favour the dominant class, classical classifier accuracy might be deceptive when there is a class imbalance. To significantly increase the performance metrics, SMOTE is utilized to rectify this imbalance. The augmentation provided a significant improvement in recall, accuracy, precision, and F1-score in the given scenario. SMOTE has a favourable influence on the classifier's capacity to successfully collect instances from the minority class, as demonstrated by the visual depiction of these metrics using bar plots. This finding emphasizes the value of using methods such as SMOTE to reduce class imbalance and create more robust and accurate predictive models in the field of heart disease.

## Conclusion

A bio-inspired HMSI model with an AttGRUnetwork is proposed in this paper for the prediction of heart disease, a life-threatening complication that can lead to heart attack and death. The algorithms' procedures are designed within the big data platform of Apache Hadoop. First, IKC eliminates outliers from the reprocessed medical data, and SMOTE balances the distribution of classes.Next, RFE selects the most significant features and model training employs HMSI to enhance the weight and bias in the AttGRU network. The proposed AttGRU-HMSI method correctly predicts cardiac disease with an accuracy of 95.42%, precision of 92.51%, recall of 98.86%, and an F1-score of 95.58%, suggesting the approach is practical. Itcan be trusted in the prediction of heart disease.Additional efforts and resources are required to collect more observational data with extensive follow-ups to develop population-specific models that solve all the issues of existing risk prediction models. Future risk predictions are based on customized population-specific models using more advanced data and methodology. Although the results were significant amongst classifiers, the general pattern showed that using RFE in feature selection can improve the discriminative ability of machine learning models when it comes to heart disease prediction. More intelligent feature selection techniques catered to specificclassifiers and datasets are made possible by the subtle insights gleaned from this comparison research. As per future scope it can be used in Investigate the integration of QANA with feature selection and ensemble learning methods to improve diagnostic accuracy and interpretability in diverse heart disease subtypes. It can Develop a hybrid AI system combining QANA and medical imaging analysis for personalized risk prediction and early detection of heart disease.

## Data Availability

The dataset analysed during the current study is Cleveland Heart Disease Dataset. [Online]. Available: https://archive.ics.uci.edu/dataset/45/heart+disease, Last Accessed: [13 DECEMBER 2023].
